# Rapid diagnosis of *Mycobacterium marinum* infection using targeted nanopore sequencing: a case report

**DOI:** 10.3389/fcimb.2023.1238872

**Published:** 2023-10-30

**Authors:** Yan-Ying Huang, Qiu-Shi Li, Zhao-Dong Li, Ai-Hua Sun, Sheng-Ping Hu

**Affiliations:** ^1^ Department of Pathology, Hangzhou Red Cross Hospital, Hangzhou, China; ^2^ Department of Ophthalmology, Hangzhou Red Cross Hospital, Hangzhou, China; ^3^ Department of Clinical laboratory, Hangzhou Red Cross Hospital, Hangzhou, China; ^4^ School of Basic Medical Sciences and Forensic Medicine, Hangzhou Medical College, Hangzhou, China; ^5^ Department of Orthopaedic, Hangzhou Red Cross Hospital, Hangzhou, China

**Keywords:** *Mycobacterium marinum*, NTM, nanopore sequencing, MinION, third generation sequencing

## Abstract

*Mycobacterium marinum* (*M. marinum*) is a non-tuberculous *mycobacterium* (NTM) that can cause infectious diseases in aquatic animals and humans. Culture-based pathogen detection is the gold standard for diagnosing NTM infection. However, this method is time-consuming and has low positivity rates for fastidious organisms. Oxford Nanopore MinION sequencing is an emerging third-generation sequencing technology that can sequence DNA or RNA directly in a culture-independent manner and offers rapid microbial identification. Further benefits include low cost, short turnaround time, long read lengths, and small equipment size. Nanopore sequencing plays a crucial role in assessing drug resistance, clinical identification of microbes, and monitoring infectious diseases. Some reports on *Mycobacterium tuberculosis* (MTB) using nanopore sequencing have been published, however, there are few reports on NTM, such as *M. marinum*. Here, we report the use of nanopore sequencing for the diagnosis of *M. marinum*.

## 1 Introduction

*Mycobacterium marinum* (*M. marinum*) is a slow-growing, non-tuberculous *mycobacterium* (NTM) and the leading cause of extra-respiratory NTM infections worldwide. It was first isolated from fish in 1926 (Streit et al., 2006; Ackleh et al., 2015). The first human case of *M. marinum* infection was reported in 1951 by Norden and Linell (Hashish et al., 2018), the infection presents as a nodular granulomatous disease (Johnson and Stout, 2015)*. M. marinum* culture is time-consuming and has a low positivity rate (70%) (Yu et al., 2023) for pathogen detection. *M. marinum* is a photochromogen that produces yellow pigment after exposure to light. Ziehl-Neelsen staining of specimens is rarely positive (Akram and Aboobacker, 2022), and even positive smear microscopy analysis cannot distinguish *M. marinum* from other mycobacteria (Aubry et al., 2017). Traditionally, *M. marinum* infections have been diagnosed in the laboratory using culture-based detection which requires a significant amount of time to isolate the bacterium from the clinical specimens and identify the species. *M. marinum* grows best at 30 °C but is inhibited at 37 °C (Aubry et al., 2017). Therefore, microscopic and culture-based results are often negative in clinical microbiological studies. PCR and serological assays are rapid and culture-independent techniques; however, both require prior knowledge of the types of pathogenic microorganisms. Moreover, these methods focus on individual pathogens rather than entire populations (Rajapaksha et al., 2019). Fortunately, recent technological advances in nanopore sequencing by Oxford Nanopore Technologies (ONT) have the potential to address these challenges.

Pocket-sized MinION was the first commercially available third-generation nanopore sequencing device developed by ONT in 2014 (Reuter et al., 2015). which is powered directly by a USB port from a laptop computer. The basic working principle of nanopore sequencing is to monitor electrical current changes caused by nucleic acids passing through a nanopore protein. Subsequently, the resulting signals are decoded to provide a specific DNA or RNA sequence (Wang et al., 2015). This new sequencing technology has many advantages, including short sequencing time, long read length, and low cost (Walter et al., 2017; Taylor et al., 2019a). The maximum turnaround time from sample collection to the delivery of the results is 6 h (Greninger et al., 2015), with data acquisition completed within 10 min once the sample is loaded onto the MinION (Greninger et al., 2015). Because of its portability, a MinION nanopore sequencer can be taken directly to the patient’s bedside and used to detect pathogens in many different environments (Reuter et al., 2015). In addition, nanopore sequencing is becoming more clinically viable owing to continued improvements in its accuracy and throughput. This technology has made nanopore sequencing an attractive diagnostic technique that produces longer reads to improve the clinical diagnosis, research, and epidemiological tracking of infectious diseases. Many studies have reported on the analysis of *Mycobacterium tuberculosis* (MTB) using nanopore sequencing (Smith et al., 2020; Gliddon et al., 2021; Dippenaar et al., 2022); however, there are few reports on NTM, such as *M. marinum* (Xing et al., 2022). Here, we report the use of third-generation nanopore sequencing to diagnose a case of *M. marinum*.

## 2 Case description

### 2.1 Patient and clinical findings

**Figure 1:**
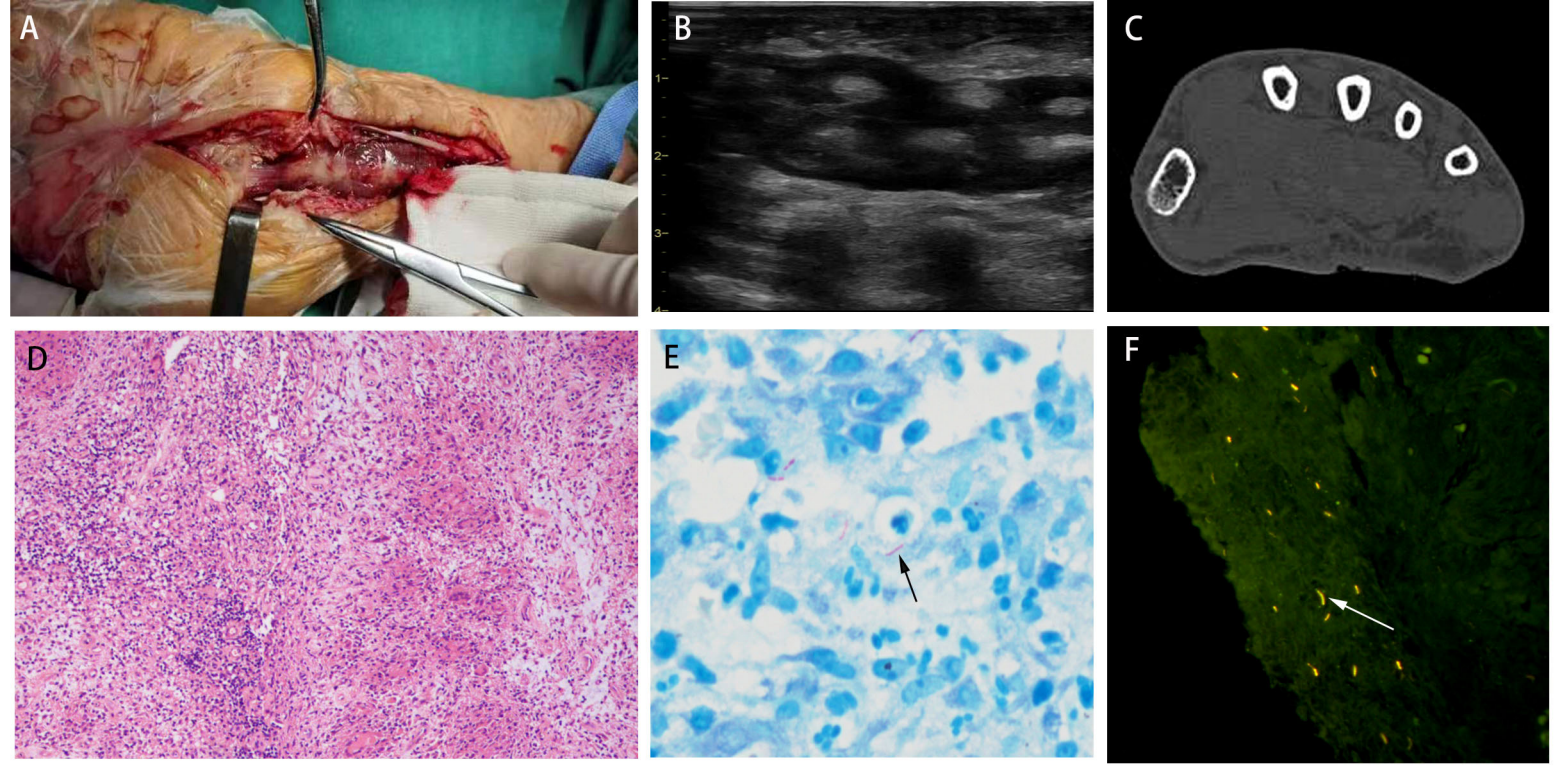
**(A)** The left wrist was wrapped with yellow granulation and extensive, localized yellow purulent discharge. **(B)** B-scan ultrasonography revealed infectious lesions in the flexor tendon sheaths of the left lower forearm, left wrist, and left index finger. **(C)** Computed tomography of the left wrist showed fluid accumulation around the tendons of the thumb and flexor carpi. **(D)** Pathological examination revealed chronic granulomatous inflammation (H&E; ×200). **(E)** Ziehl-Neelsen staining for AFB was positive. **(F)** The fluorescence staining for AFB was strongly positive.

A 67-year-old man was admitted with a history of chronic pain in the left hand that had reduced his mobility and dexterity for more than four months. The initial injury had occurred when a crab bit his left index finger. Topical disinfection was not performed. This resulted in the spread of the infection and severe swelling of the left palm and wrist. Resection of the wrist lesion revealed that finger flexor tendons 1-5 were covered with yellow granulation and an extensive, localized yellow purulent discharge (**Figure 1A**). The focal tissue of the left wrist was excised and sent for pathological examination. B-scan ultrasonography revealed infectious lesions in the flexor tendon sheaths of the left lower forearm, left wrist, and left index finger (**Figure 1B**). Computed tomography of the left wrist showed fluid accumulation around the tendons of the thumb and flexor carpi; therefore, tenosynovitis was suspected (**Figure 1C**). Pathological examination revealed chronic granulomatous inflammation with small necrotic foci (**Figure 1D**). Routine laboratory tests yielded negative results for MTB and NTM DNA. No mycobacterial growth was observed after 42 days of culture. Ziehl-Neelsen staining for AFB was positive (**Figure 1E**). The fluorescence staining for AFB was strongly positive (**Figure 1F**). Therefore, it was necessary to further distinguish between NTM and MTB.

### 2.2 Nanopore sequencing

**Figure 2:**
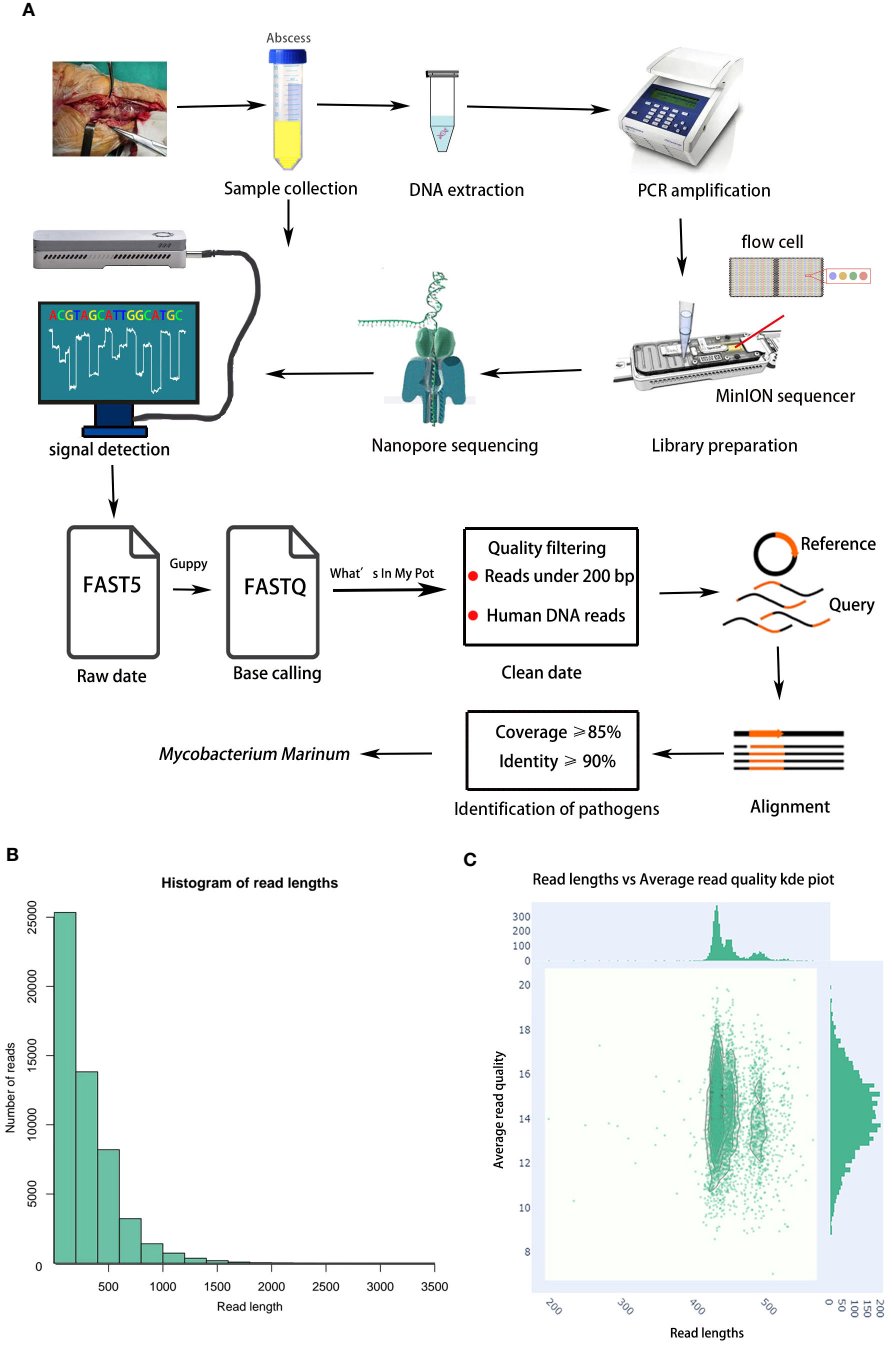
**(A)** Flow chart of nanopore sequencing. An abscess sample was collected in a sterile tube, DNA from sample was extracted. The extracted DNA was used for PCR amplification. The PCR products were used for library preparation. The purified libraries were loaded onto a flow cell on MinION platform. The Guppy convert FAST5 files to FASTQ for data analysis. Reads under 200 bp and Human DNA reads were filtered. Sequenced reads were analyzed by the What’s In My Pot workflow via EPI2ME. Finally, the remaining reads were aligned to the NCBI Microorganism Genome Database. **(B)** The read length distribution histogram. **(C)** Quality analysis of MinION sequencing.

To further characterize the pathogen, nanopore MinION sequencing was performed to accurately identify the infectious agent. The protocol for using MinION is described below and shown in **Figure 2A**.

#### 2.2.1 Sample collection

First, an abscess sample was collected from the patient’s left hand into a sterile tube. The samples met the criteria for clinical examination. The samples were processed immediately, unless otherwise specified.

#### 2.2.2 DNA extraction and amplification

The sample and an equal volume of DTT solution were digested with 10 μLproteinase K and 5 μL lysozyme and ground using 0.05 mm zirconia grinding beads. The grinding was performed using a grinder. The nucleic acid was extracted from the ground sample with magnetic beads using the QIAamp DNA Microbiome Kit (Cat. No.51707, Qiagen, Hilden, Germany) according to the manufacturer’s protocol. The concentration of the extracted DNA was determined. PCR amplification was performed to detect bacterial 16S rRNA genes. The primers used for PCR amplification are listed in **Supplementary Table 1**. PCR amplification was performed using an ABI 2720 Thermal Cycler (Cat. No. 435659; ABI, California, CA, USA) under the following conditions: an initiation denaturation step at 95°C× 3 min, then six cycles at 95°C × 30 s/64°C × 30 s/72°C × 60 s, and a final extension step at 72°C × 3 min. The PCR amplification products were ranged from 200 to 850 bp. The PCR amplification products were purified and quantified using Qubit 4 and agarose gel electrophoresis for subsequent library preparation and nanopore sequencing.

#### 2.2.3 Library preparation and nanopore sequencing

The nanopore barcode PCR products were purified with 0.6 × AMPure beads, and each purified barcode PCR product was pooled in equal amounts for nanopore library preparation. which was constructed using a ligation sequencing kit (Cat. No. SQK-LSK109, Oxford Nanopore Technologies, Oxford, UK) according to the manufacturer’s instructions. Next, 100ng of the final prepared library was loaded onto a flow cell (R9.4.1) and inserted into a sequencer connected to a computer. The GridION platform was used for sequencing, and MinKNOW software was used to output the base-calling data. Barcode demultiplexing was performed using Porechop (version. 0.2.4). During MinION sequencing, the current signal as the nucleic acid molecules passed through the nanopore was detected, and the data were stored in a FAST5 format file. Then, Guppy (version 3.2.1, ONT, Oxford, UK) was used to convert FAST5 files to FASTQ for data analysis.

#### 2.2.4 Pathogen identification

First, quality filtering of original sequencing reads was performed, followed by follow-up analysis and sequencing reads for bacterial classification were analyzed using the What’s In My Pot (WIMP) workflow via the EPI2ME platform (version 3.2.2, ONT, Oxford, UK). EPI2ME is a set of real-time analytical tools that includes species identification and reads quality control. Reads of < 200 bp were filtered. Human DNA reads were removed via alignment with the human reference genome. Finally, the remaining reads were aligned to the NCBI Microorganism Genome Database. Pathogens were classified at the species level based on percentage coverage and identity. Generally, the top 10 microorganisms, ranked by aligned reads and with a relative abundance score > 0.5%, were classified as pathogens and subjected to further evaluation. Potential pathogen(s) were reported if the number of reads accounted for ≥ 1% of microbial reads and had a WIMP alignment score ≥ 20 (Charalampous et al., 2018). The time required for identification was 16–17 h from the start of the test to the delivery of the test results. The quality filter was Q7. A total of 19,872 reads were obtained, including 16,918 high-quality reads; the average length of all reads was 2686 bp (range: 200 to 3554 bp, **Figures 2B, C**). The *M. marinum* sequence (accession:GCA_000723425.2) was ultimately used for identification. The positive threshold of effective reads for analysis was set to coverage ≥ 85% and identity ≥ 90%. Finally, nanopore sequencing detected *M. marinum* in purulent secretion samples. The number of reads was 4176 (**Supplementary Table 2**), and the relative abundance was 92.37%. The patient received combination therapy with ethambutol (750 mg/day, oral), rifampin (450 mg/day, oral), and isoniazid (300 mg/day, oral). Eventually, he experienced full resolution of his symptoms, and no relapse was observed during the six-month follow-up period.

## 3 Discussion

Both NTM and MTB belong to the genus *Mycobacterium* and have similar pathogenic mechanisms and clinical manifestations, which often result in misdiagnosis. Culture-based detection is a conventional method for diagnosing NTM infections. However, *M. marinum* is a slow-growing microorganism that is difficult to culture. Typically, it takes over two weeks to grow, and optimal growth requires low temperatures (approx. 30 °C) (Aubry et al., 2017). The isolation culture-based detection is generally performed at 37°C; therefore, a risk of culture-negative infections misdiagnosis emerges. In addition, *M. marinum* and *M. ulcerans* share more than 98% of their genomes, and no current PCR techniques can distinguish between them (Aubry et al., 2017). Next generation sequencing (NGS) platforms can identify all microorganisms in a sample within 24 h (Naccache et al., 2014; van Dijk et al., 2014). However, short sequencing reads make it difficult to parse the complex genomic structures of microorganisms. In addition, the high cost, lengthy and tedious processes, and large equipment size remain major deterrents to its routine use in clinical settings. Therefore, there is an urgent need to develop rapid and accurate methods to detect pathogenic microorganisms. Nanopore sequencing is increasingly recognized for its advantageous characteristics, including short turnaround time (< 6 h), long read lengths, small equipment size, and affordability, making it a valuable asset in a clinical microbiology laboratory (Jain et al., 2016; Petersen et al., 2019). Recently nanopore sequencing has enabled the sequencing of bacterial, viral, and fungal genomes (Ashton et al., 2015; Loman et al., 2015; Istace et al., 2017).

In our case, the patient had abscesses with yellow pus and granulomas that were visible on pathological examination, suggesting a possible MTB or NTM infection. Ziehl-Neelsen and fluorescent staining for AFB were positive. However, microscopy alone cannot distinguish between cases of MTB and NTM, and it was difficult to determine which microorganism was responsible for the primary infections. MTB DNA and NTM DNA tests were negative in our study. No microorganisms were observed to grow in the culture medium. Nanopore sequencing was used to identify the infectious agent accurately. Fortunately, nanopore sequencing confirmed that the infection was caused by *M. marinum.* These findings indicate that Nanopore MinION sequencing can be used as a rapid diagnostic tool for infectious diseases.

Nanopore sequencing is a valuable sequencing technology, in which different nucleotide bases are distinguished by changes in the current when an ionic current is passes across the flow cell during sequencing (Laver et al., 2015). High-throughput sequencing is used in various clinical fields, such as identification, characterization, and surveillance of pathogenic microorganisms; detection of drug resistance genes and evaluation of the resistance phenotype; and description of disease-related microbial community (Zhang et al., 2022). This method offers many solutions to the current challenges in genome sequencing. However, a major limitation of this approach is its read accuracy, which can be compromised by a high error rate and insufficient sequencing depth when the technology is used repeatedly on the same sequence (Petersen et al., 2019; Taylor et al., 2019a). In addition, there is no genus-level identification of an organism, and the utility and diagnostic yield of metagenomic sequencing is considerably reduced in other clinical situations. Most software developed to interpret nanopore sequences requires relatively high bioinformatics skills, which are unavailable to most biologists. With continued improvements in the accuracy and throughput of nanopore sequencing platforms, the sequencing error rate has reached a clinically acceptable range (Taylor et al., 2019b). In conclusion, nanopore sequencing is a reliable method for the rapid detection of pathogens in suspected infections. We believe that nanopore sequencing will become a routine diagnostic tool in the field of clinical infectious disease diagnosis and healthcare surveillance in the near future.

## Data availability statement

The datasets presented in this study can be found in online repositories. The name of the repository and accession number can be found below: https://www.ncbi.nlm.nih.gov/bioproject/?term=PRJNA967818.

## Ethics statement

This study was approved by the Ethics Committee of the Hangzhou Red Cross Hospital. The studies were conducted in accordance with the local legislation and institutional requirements. Written informed consent was obtained from the individuals for the publication of any potentially identifiable images or data included in this article. Written informed consent was obtained from the participant/patient(s) for the publication of this case report.

## Author contributions

Y-YH wrote the manuscript. S-PH and A-HS reviewed and edited the manuscript. Q-SL and Z-DL reviewed the clinical data. Y-YH and Q-SL supervised the microbiological investigations. Z-DL and S-PH reviewed the pathological data. All authors contributed to the article and approved the submitted version.
