# Supplementary material for: Rapid diagnosis of Mycobacterium marinum infection using targeted nanopore sequencing: a case report
Source: Front Cell Infect Microbiol. 2023 Oct 30;13:1238872. doi: 10.3389/fcimb.2023.1238872 (PMC10642934; doi:10.3389/fcimb.2023.1238872)
Supplement: Supplementary file 1 [file Table_1.doc]

**Supplementary Table 1 Primers details used for PCR amplification**

| **Items** | **Primer sequence** |
| --- | --- |
| Nano-16S-F | TTCCCTTGTGGCCTGTGTGCA |
| Nano-16S-R | GATCCCACCTTCGACAGCTCC |
| Nano-F | AATACCTGGTCCGCTTGCACG |
| Nano-R | GGTCTCGATCGGGCACATCCG |
