# Supplementary material for: Rapid diagnosis of Mycobacterium marinum infection using targeted nanopore sequencing: a case report
Source: Front Cell Infect Microbiol. 2023 Oct 30;13:1238872. doi: 10.3389/fcimb.2023.1238872 (PMC10642934; doi:10.3389/fcimb.2023.1238872)
Supplement: Supplementary file 2 [file Table_2.doc]

**Supplementary table 2 NanoPlot reports about statistics**

| **General summary** |  |
| --- | --- |
| Mean read length | 450.8 |
| Mean read quality | 13.3 |
| Median read length | 441.0 |
| Median read quality | 14.2 |
| Number of reads | 4,176.0 |
| Read length N50 | 442.0 |
| STDEV read length | 27.0 |
| Total bases | 1,882,406.0 |
| **Number, percentage and megabases of reads above quality cutoffs** |  |
| >Q5 | 4176 (100.0%) 1.9Mb |
| >Q7 | 4176 (100.0%) 1.9Mb |
| >Q10 | 4128 (98.9%) 1.9Mb |
| >Q12 | 3706 (88.7%) 1.7Mb |
| >Q15 | 1416 (33.9%) 0.6Mb |
| **Top 5 highest mean basecall quality scores and their read lengths** |  |
| 1 | 20.2 (543) |
| 2 | 19.9 (435) |
| 3 | 19.8 (449) |
| 4 | 19.7 (451) |
| 5 | 19.4 (429) |
| **Top 5 longest reads and their mean basecall quality score** |  |
| 1 | 569 (13.1) |
| 2 | 561 (15.0) |
| 3 | 559 (11.2) |
| 4 | 555 (11.9) |
| 5 | 552 (12.3) |
